# Effectiveness of Radiomic ZOT Features in the Automated Discrimination of Oncocytoma from Clear Cell Renal Cancer

**DOI:** 10.3390/jpm13030478

**Published:** 2023-03-06

**Authors:** Gianluca Carlini, Caterina Gaudiano, Rita Golfieri, Nico Curti, Riccardo Biondi, Lorenzo Bianchi, Riccardo Schiavina, Francesca Giunchi, Lorenzo Faggioni, Enrico Giampieri, Alessandra Merlotti, Daniele Dall’Olio, Claudia Sala, Sara Pandolfi, Daniel Remondini, Arianna Rustici, Luigi Vincenzo Pastore, Leonardo Scarpetti, Barbara Bortolani, Laura Cercenelli, Eugenio Brunocilla, Emanuela Marcelli, Francesca Coppola, Gastone Castellani

**Affiliations:** 1 Department of Physics and Astronomy, University of Bologna, 40127 Bologna, Italy; 2 Department of Radiology, IRCCS Azienda Ospedaliera-Universitaria di Bologna, 40138 Bologna, Italy; 3 eDIMESLab, Department of Medical and Surgical Sciences, University of Bologna, 40138 Bologna, Italy; 4 Department of Medical and Surgical Sciences, University of Bologna, 40138 Bologna, Italy; 5 Division of Urology, IRCCS Azienda Ospedaliero-Universitaria di Bologna, 40138 Bologna, Italy; 6 Department of Pathology, IRCCS Azienda Ospedaliero-Universitaria di Bologna, 40138 Bologna, Italy; 7 Department of Translational Research, Academic Radiology, University of Pisa, 56126 Roma, Italy; 8 National Institute of Nuclear Physics, INFN, 40127 Bologna, Italy; 9 Department of Biomedical and Neuromotor Sciences, University of Bologna, 40138 Bologna, Italy; 10 Dipartimento Diagnostica per Immagini AUSL Romagna, UOC Radiologia Faenza, 48018 Faenza, Italy; 11 Italian Society of Medical and Interventional Radiology, SIRM Foundation, 40138 Bologna, Italy

**Keywords:** computer-aided diagnosis, image analysis, radiomic, machine learning, renal oncocytoma, renal cell carcinoma, small renal masses, computed tomography

## Abstract

Background: Benign renal tumors, such as renal oncocytoma (RO), can be erroneously diagnosed as malignant renal cell carcinomas (RCC), because of their similar imaging features. Computer-aided systems leveraging radiomic features can be used to better discriminate benign renal tumors from the malignant ones. The purpose of this work was to build a machine learning model to distinguish RO from clear cell RCC (ccRCC). Method: We collected CT images of 77 patients, with 30 cases of RO (39%) and 47 cases of ccRCC (61%). Radiomic features were extracted both from the tumor volumes identified by the clinicians and from the tumor’s zone of transition (ZOT). We used a genetic algorithm to perform feature selection, identifying the most descriptive set of features for the tumor classification. We built a decision tree classifier to distinguish between ROs and ccRCCs. We proposed two versions of the pipeline: in the first one, the feature selection was performed before the splitting of the data, while in the second one, the feature selection was performed after, i.e., on the training data only. We evaluated the efficiency of the two pipelines in cancer classification. Results: The ZOT features were found to be the most predictive by the genetic algorithm. The pipeline with the feature selection performed on the whole dataset obtained an average ROC AUC score of 0.87 ± 0.09. The second pipeline, in which the feature selection was performed on the training data only, obtained an average ROC AUC score of 0.62 ± 0.17. Conclusions: The obtained results confirm the efficiency of ZOT radiomic features in capturing the renal tumor characteristics. We showed that there is a significant difference in the performances of the two proposed pipelines, highlighting how some already published radiomic analyses could be too optimistic about the real generalization capabilities of the models.

## 1. Introduction

Renal cell carcinoma (RCC) affects more than 400,000 individuals per year worldwide [1]. Clear cell RCC (ccRCC) is the most common histotype of RCC, making up approximately 70% of the total cases [2]. The widespread use of cancer screening imaging increased the detection of incidental small renal masses (≤4 cm), a non-negligible rate of which (ranging from 7 to 33%) is benign lesions, such as renal oncocytoma (RO) [3]. The proper management of renal masses is highly influenced by the efficacy of diagnostic radiology. Therefore, the distinction between ccRCC and RO is the most intriguing diagnostic challenge.

Routine clinical imaging techniques, such as contrast-enhanced computed tomography (CT) and magnetic resonance imaging (MRI), cannot completely distinguish between malignant RCC and benign renal tumors. Particularly, many studies have attempted to distinguish ccRCC from oncocytoma on the basis of the analysis of qualitative and quantitative parameters derived from multiphase high-resolution CT [4,5,6,7,8,9], but no morphological characteristics or angiodynamic behaviors after contrast medium have been shown to be able to definitively distinguish ccRCC from RO. Both these lesions, in fact, appear hyper-vascularized in the arterial phase and without distinctive features, especially when small in size.

The emerging automated solutions provided by the introduction of artificial intelligence algorithms, based their efficiency on the analysis of radiomic features. With the term radiomic, we identify the quantitative analysis of a set of features extracted from medical images, including CT, positron emission tomography (PET), and MRI. Radiomic features can be used to build machine-learning-aided models providing valuable diagnostic, prognostic, and predictive information [10,11,12]. In the last few years, radiomic features were revealed to be promising predictors in several cancer-related studies [13,14,15,16,17,18], providing quantitative scores for the characterization of tumor volumes that would otherwise be inaccessible.

Many authors have already proposed radiomic solutions for the automated analysis of renal cancer [19]. Li et al. [20] used enhanced CT radiomic features to train five machine learning models to differentiate renal chromophobe cell carcinoma from RO, obtaining ROC AUC scores higher than 0.85 with all the models tested. Wang et al. [21] trained three machine learning models on top of enhanced CT radiomic features to discriminate clear cell from non-clear-cell renal carcinomas. They compared the performance of the models with the radiologist’s diagnosis, showing that the tested models obtained equivalent or better results. Li et al. [22] built a radiomic nomogram incorporating CT radiomic features and clinical variables, showing superior capabilities in differentiating ccRCC from RO with respect to a model including clinical variables only. Baghdadi et al. [23] combined a deep-learning-based tumor segmentation technique with the computing of the peak early-phase enhancement ratio (PEER) to differentiate RO and ccRCC using CT images. Finally, Deng et al. [24] investigated the usefulness of texture analysis in differentiating RO and RCC in a retrospective study.

However, all these authors focused their analysis only on the tumor volume, neglecting the status of the surrounding areas. The information contained in the areas of transition between the tumor and the healthy tissues could provide interesting features about the pattern of proliferation of the lesion.

Moreover, the accuracy of the tumor segmentation has a fundamental role in the radiomic feature extraction and analysis. Segmenting the tumor region is challenging because tumors may have indistinct borders [25,26]. In this case, considering the surrounding region allows this effect to be reduced [27].

The tumor zone of transition (ZOT) is defined as the peripheral zone of the tumor, in which the CT attenuation is lower than the attenuation of the tumor’s solid center, but still higher than the attenuation of the surrounding non-tumoral tissue. Radiomic studies on RCC do not usually consider the tumor ZOT. However, it can provide valuable information about the tumor’s relation with the surrounding healthy parenchyma, and it can be used to better measure the tumor volume variations in subsequent follow-ups [28]. For these reasons, we included radiomic features extracted from the tumor ZOT with the ones collected from the tumor volumes.

A critical point in radiomic analyses is the approach used for the feature selection. Specifically, in some proposed radiomic pipelines, feature selection is performed before the splitting of the data into training and test sets, or, equivalently, before the cross-validation procedure. This may lead to cross-contamination of the model with information deriving from the test set, which in principle should be unknown. To investigate this aspect, we propose two different pipelines: in the first one, the feature selection is performed before the train-test split; in the second one, the feature selection is performed after, i.e., on the training set only. To give a more robust measure of the model performance, we evaluated it several times by repeating the train-test split procedure, each time shuffling the data randomly.

The aim of this study was to build a machine learning model based on CT radiomic features to discriminate RO from ccRCC, focusing on ZOT features in capturing tumor characteristics which are usually overlooked. We also analyzed the impact of performing the feature selection at different points of the radiomic pipeline, showing how some already published radiomic analyses could be too optimistic about the real generalization capabilities of the models.

## 2. Materials and Methods

### 2.1. Patient Selection

We collected data from 77 patients (39% cases of RO and 61% cases of ccRCC), with a single T1 renal mass who underwent partial nephrectomy at single tertiary urologic center from January 2019 to December 2021. Inclusion criteria were as follows: (1) a complete renal CT evaluation with a specific protocol (unenhanced, arterial, parenchymal, and excretory phases) with thin-slice reconstruction (≤2.5 mm); (2) a renal mass ≤ 4 cm at CT imaging; (3) a diagnosis of ccRCC or RO in the histopathological report from the surgical specimen by a dedicated pathologist of the Pathology Unit of our Institution. Exclusion criteria were as follows: (1) incomplete renal-CT-specific protocol; (2) previous renal surgery; (3) the presence of severe artifacts, not allowing for the evaluation of one or more CT phases. Images were retrieved from subjects who gave their voluntary consent to research. The data acquisition protocol was approved by the local ethics committee (protocol n° EM566–2021 577/2018/Sper/AOUBo approved on 8 July 2021) according to the Helsinki Declaration.

### 2.2. Image Acquisition and Radiomic Features Extraction

The images were acquired by three different helical CT scanners: (1) Somatom Emotion 6-slice (Siemens Healthcare, Erlangen, Germany) using the following parameters: voltage 120–140 kV, tube current 120 mA, collimation 6 × 2 mm, rotation 0.6–0.8 s, and pitch factor 0.85; (2) Somatom Sensation Cardiac 16-slice (Siemens Healthcare, Erlangen, Germany) using the following parameters: voltage 120–140 kV, tube current 120 mA, collimation 16 × 1.2 mm, rotation 0.6 s, and pitch factor 0.8; (3) GE VCT Light Speed 64-slice (GE Healthcare, Milwaukee, WI, USA) using the following parameters: voltage 120–140 kV, tube current 120 mA, collimation 64 × 0.625 mm, rotation 0.35 s, and pitch factor 0.85. The section collimation and interval reconstruction were 5 mm and 2.5 mm, or 2.5 mm and 1.25 mm, respectively, according to the type of scanner used. Unenhanced, arterial, parenchymal, and delayed (excretory) phases were obtained in the axial plane through the kidneys during patient breath-holding. Intravenous non-ionic contrast material (Iomeprol 300/350 mg/mL, Iomeron; Bracco Imaging srl, Milan, Italy), administered according to patient weight (range 120–140 mL), was injected at a flow rate of 3 mL/s (or slower in cases of suboptimal venous access) followed by 20 mL of saline solution. The time delay to scanning was determined based on the typical time to the renal arterial (25–30 s), parenchymal (80–100 s), and delayed (5–10 min) phases.

All 3D virtual reconstructed models were conducted by a trained technician (B.B.) at the eDIMESLab of the University of Bologna, and by radiologists of the IRCCS, Azienda Ospedaliero-Universitaria, S. Orsola-Malpighi Hospital. Briefly, the acquired CT DICOM data of arterial, parenchymal, and excretory phases were used for the selective identification of the tumor lesion in the image segmentation process, although the next radiomic analyses were conducted on the arterial phase only. Segmentation was achieved using D2P^TM^ (‘DICOM to PRINT’; 3D Systems Inc., Rock Hill, SC, USA), which is a certified software to convert DICOM medical images into 3D digital models. The segmentation results and the correctness of the reconstructed 3D virtual models were reviewed and validated by experienced radiologists (C.G. and A.R.) as previously described [29,30,31].

For each patient, a binary mask related to the tumor volume(s) was identified and stored. The obtained volume segmentations were used as inputs for radiomic feature extraction, including the 2D, 3D, Laplacian, and wavelet standard features, for a total of 1218 features.

**Figure 1:**
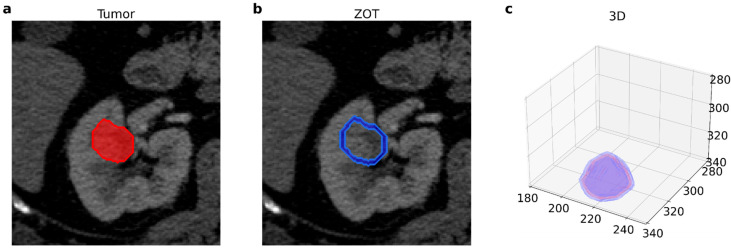
Example of tumor volume and related ZOT volume used in the current pipeline. (**a**) Tumor volume manually identified by the experts. (**b**) ZOT volume automatically extracted starting from the identified tumor volume: the ZOT takes care of the volume inside and surrounding the tumor. The ZOT is automatically extracted via a morphological algorithm applied on the tumor mask. (**c**) 3D representation of the two volumes: the red surface (tumor) is encapsulated between the two blue concentric surfaces (ZOT).

We identified the ZOT using morphological operations, as described in the literature [28,32,33]: starting from each manually identified tumor volume, we applied two times morphological 3D operations (dilation and erosion) with a circular kernel of size 3 × 3 × 3 voxels, obtaining eroded and dilated binary volumes. The ZOT was obtained by subtracting the eroded volume from the dilated one. An example of tumor segmentation and the related ZOT volume is shown in Figure 1. The same set of radiomic features (1218 features) was extracted from the ZOT areas, obtaining a total of 2436 radiomic features for each patient. The entire set of features was extracted using the Pyradiomics library [34].

### 2.3. Feature Selection

We used a genetic algorithm (GA), following the method presented by Carlini et al. [14], to select the most predictive subset of features. Each genome identified a putative subset of features, expressed as a binary pattern of the total number of features. Features associated with no-null genes were preserved, while those associated with null genes were excluded. Each genome was assessed using a decision tree classifier (DTC). The DTCs built with the selected feature subsets were evaluated using a metric function and the scores of the models were used as fitness values of the genomes. The metric function was the average area under the receiver operating characteristic curve (ROC AUC) obtained in a five-fold cross-validation. In this way, the best genome, i.e., the one selecting the best features, was the one producing the classifier which maximized the ROC AUC score in the five-fold cross-validation. The genomes were initialized randomly; then, as the generations continued, they became more and more specialized in selecting the best features by maximizing the fitness function. In this feature selection pipeline, 150 genomes were evaluated for a total of 100 generations.

### 2.4. Classification Model

The best selected features were used to build a DTC. To evaluate the classifier, we split the initial dataset into two mutually exclusive subsets: a training set containing 80% of the data, and a test set containing the remaining 20%. We stratified the data on the basis of the outcome label to ensure an equal proportion of the two classes of patients between the train and test sets. Here we present two versions of the pipeline. In the first version, the feature selection was performed before the splitting of the data, i.e., on the whole dataset. In the second version, instead, the feature selection was performed after the splitting of the data, i.e., on the training data only. We did so to evaluate the impact of performing feature selection at different points in the pipeline. Some radiomic analyses proposed in the literature, indeed, perform the feature selection before the splitting of the data. However, in this way, the model can be cross-contaminated with information deriving from the test data, which should be unknown. This could lead to an overestimation of the actual model performance and generalization capabilities.

For both the pipelines, we repeated the entire process of feature selection, splitting of the data, and classifier validation 100 times; each time we shuffled the data randomly before splitting them into training and test sets. In this way, we could measure the average score of the classifier across the different data splitting, obtaining a more robust estimation of the model performance. In principle, the repetition of the feature selection step should not be needed if it is applied to the whole dataset, since the data does not change. However, the GA is stochastic in nature, so it will identify different optimal feature subsets if it is executed multiple times on the same data. We used the ROC AUC as the scoring function.

**Figure 2:**
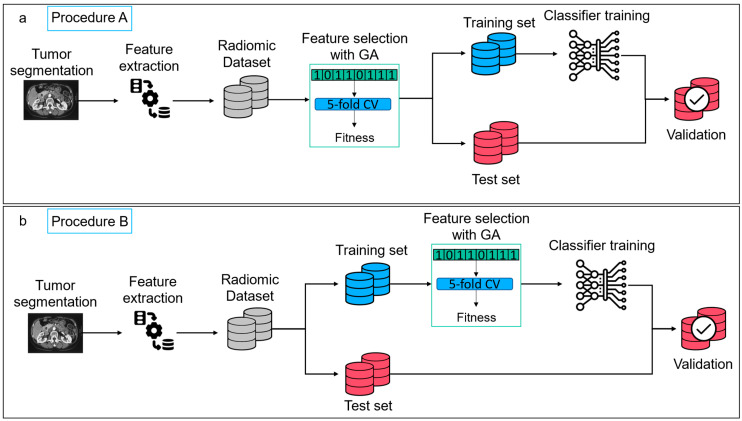
Scheme of the two proposed pipelines. Starting from the raw patient CT and the manually identified tumor volume and ZOT, a set of 2436 radiomic features was extracted. (**a**). A feature selection via GA was performed on the entire dataset of features (**Procedure A**), then, 80% of the data was used for the training of a DTC and the remaining 20% was used for testing the model performance. (**b**). Second version of the radiomic pipeline (**Procedure B**), where the feature selection was performed before the train-test splitting, i.e., on the training dataset only.

With the repetition of the evaluation procedure several times, we could also estimate the feature importance by calculating how many times the same feature was selected. We monitored the radiomic features extracted by the GA application on both the pipeline schemes, across the 100 random subdivisions. For each GA application, we considered the top performing genome and the related features subset identified. We evaluated the number of times in which the same feature was selected and the related selection rate across the 100 simulations. The scheme of the two proposed pipelines is shown in Figure 2.

## 3. Results

**Figure 3:**
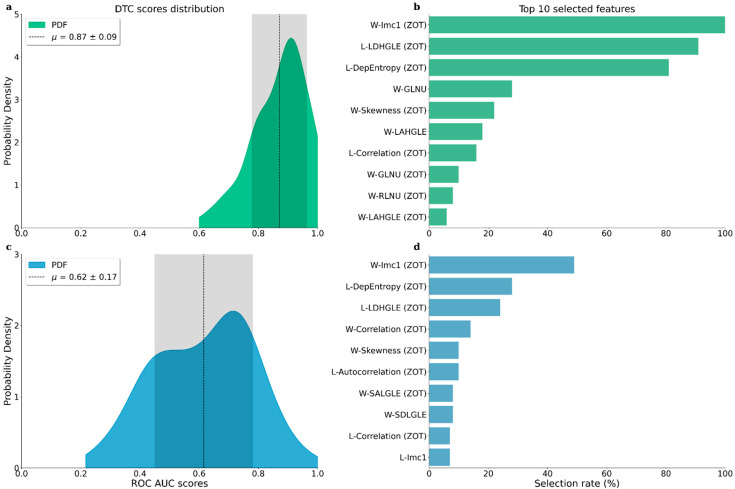
Results obtained by the two proposed radiomic pipelines. (**a**–**c**) Probability density distributions of ROC AUC scores obtained by the DTC classifiers on 100 train-test splitting, following the scheme of Procedure A and Procedure B, respectively. We highlighted with the dashed line the means of the distributions and with the shadowed areas the corresponding standard deviations. (**b**–**d**) Top 10 radiomic features selected by the GA, following the scheme of Procedure A and Procedure B, respectively. For each feature, we highlighted the selection rate (%) obtained during the 100 train-test subdivisions performed. We reported with an initial W- and L- the radiomic features related to the wavelet and Laplacian transformations, respectively. The features extracted from the ZOT areas were highlighted with a final (ZOT) keyword.

The distribution of the results obtained by the application of *Procedure A* and *Procedure B*, repeated for 100 random subdivisions of the data, are reported in Figure 3a and Figure 3c, respectively.

We reported in Figure 3b,d the top 10 selected radiomic features obtained by the application of *Procedure A* and *Procedure B*, respectively. We highlighted in the figures the radiomic features related to the ZOT areas, distinguishing them from the tumor volume ones.

The pipeline proposed in *Procedure A* obtained an average ROC AUC score of 0.87, with a standard deviation of 0.09, in agreement with other state-of-the-art studies on RCC [21,35,36]. The scores obtained with the pipeline proposed in *Procedure B* were generally lower, with an average ROC AUC score of 0.62 and a standard deviation of 0.17. However, they are still compatible with state-of-the-art results [37]. Both the pipelines achieved a ROC AUC score higher than 0.5, showing that CT radiomic features can be successfully used to build machine learning models capable of discriminating ccRCC patients from RO patients.

## 4. Discussion

The proposed *Procedure A* obtained a ROC AUC of 0.87 ± 0.09. This result agrees with the state-of-the-art ones: Wang et al. [21] proposed three different classifiers, achieving a ROC AUC of 0.909, 0.841, and 0.906, using only radiomic features from the tumor region from 190 patients. Similarly, Chen et al. [35], proposed three different models considering different contrast-enhanced computed tomography (CECT) phases, achieving a ROC AUC of 0.748–0.823, 0.776–0.887, and 0.864–0.900, respectively. Finally, Van Oostenbrugge et al. [36], proposed a single model, reaching a ROC AUC of 0.86, training and validating the model on 39 patients. The scores obtained by *Procedure B* were generally lower, with an average ROC AUC of 0.62±0.17. However, this result is still comparable with the state-of-the-art ones, as in the work of Gao et al. [37]. In their work, Gao et al. proposed a combination of nomogram, radiomic, and clinicopathological signatures, reaching a ROC AUC of 0.768 on the validation cohort. We showed the importance of the feature selection process; in particular, we highlighted the impact on the model’s performance when applied at different points in the pipeline. The two pipelines we proposed obtained remarkably different results, with *Procedure A* reaching an average score that was 25% higher than the one achieved in *Procedure B*. The main difference in the two procedures was that in *Procedure A*, the feature selection is done on the entire dataset, while in *Procedure B*, it is performed after splitting the data on the training set only. This shows how performing feature selection before the splitting of the data, i.e., on the whole dataset, can introduce cross-contamination between the training and the test sets, leading to a higher measured model performance. The performance obtained in this way, however, may not accurately represent the actual generalization capabilities of the model. This phenomenon is known as overfitting and should be carefully considered when performing radiomic analyses, especially since the radiomic data samples are relatively small, so the effect of overfitting is even more prominent.

Our findings confirm that radiomic features can provide valuable information about complex tumor features that would otherwise be inaccessible. In particular, our results show that CT-derived radiomic features can capture the subtle differences between ccRCC and RO, allowing for the successful discrimination of the two histotypes. These differences usually cannot be acknowledged with routine clinical imaging techniques. Thus, radiomic analyses, as the one presented in this work, can be a valid non-invasive instrument to assist clinicians in clinical practice. Indeed, radiomic analysis may improve the predictive accuracy of conventional imaging to discriminate between RO and ccRCC with important clinic implications. To note, the clinical management of a T1a renal mass is elective surgery with nephron-sparing surgery whenever feasible [38,39]. However, in the case of elderly patients or in severe comorbidity, active surveillance of the mass may be an alternative approach. The discrimination of RO from ccRCC by non-invasive radiomic methods would allow for better selection of patients for active surveillance of a renal mass (i.e., in the case of high suspicion of RO) or active treatment including novel percutaneous ablation as well as surgery (in the case of high suspicion of ccRCC) [40,41].

The repetition of the train-test split allowed a more robust estimation of the classifier performance. We deem that the repetition of the model evaluation with different data splitting should be considered a good practice, especially when the sample number is small. The repeated process also allowed us to better estimate feature importance by calculating how many times the same feature was selected by the GA. The feature importance result is more interesting in the case of *Procedure B*, because the feature selection was performed each time on a different set of data, and therefore it was more heterogeneous. In *Procedure A*, instead, feature selection was performed always on the same data, so the only source of variability was given by the stochastic nature of the GA selection process. In both cases, however, eight of the top ten selected features were ZOT features, indicating that they can have an important role in discriminating RO from ccRCC. For this reason, we surmise that the usage of ZOT features should be further investigated also in other radiomic analyses. Indeed, the ZOT expresses the close relationship between neoplastic lesions and the surrounding normal parenchyma; therefore, the analysis of the ZOT can be very important in the evaluation of neoplastic lesions. Both ROs and ccRCCs are expansive lesions characterized by a different growth pattern and, consequently, a different response of the surrounding normal parenchyma. ccRCCs usually grow faster than ROs and compress the surrounding normal parenchyma forming a more evident “pseudocapsule”. By contrast, RO displays a direct interface with the surrounding parenchyma, without a real pseudocapsule, due to its slow growth. Although in this paper a radiological–pathological correlation has not been conducted, we can hypothesize that the radiomics analysis of the ZOT may be useful in distinguishing ccRCCs from ROs, highlighting the different relationships between the lesion and the surrounding parenchyma. For this reason, further studies are needed to correlate the radiomic analyses with the pathological data.

To further support the findings of this study, the application of the proposed pipelines on a more extensive dataset and the validation of the classifier on external data acquired in different medical centers would be beneficial.

## 5. Conclusions

In this work, we highlighted the importance of ZOT radiomic features in the discrimination of RO from ccRCC in a T1a renal tumor, leading to important clinical implications with regards to better selection of patients for active treatment (surgery or ablation) vs. active surveillance. Analysis of the peripheral areas of lesions allows for better monitoring of the proliferation of the tumors and their biological impact on the healthy surroundings. However, its usage and predictive capability strictly depends on the biological properties of the tumor and involved tissues. Therefore, the introduction of ZOT analysis must be clinically assessed in relation to the phenotypes of different cancers.

The splitting of the data during machine learning analysis could drastically affect the predictive results, introducing possible cross-contamination of information. More attention must be paid to this topic in radiomic analyses because it could be a source of overly optimistic evaluations and overfitted models. This consideration assumes even more importance when the available datasets are composed of small samples.

In conclusion, the results obtained by our pipelines are both in agreement with the initial assumption about the successful usage of radiomic features in discriminating ROs from ccRCC patients.

## Data Availability

The data used during the current study are available from the corresponding author on reasonable request. The pre-trained model and parameters used for the radiomic analysis are publicly available in the repository, Github-repo (https://github/GianlucaCarlini/radiomics_renal_cancer), accessed on 5 March 2023.
